# A Randomized, Single-Blind, Crossover Trial of Recovery Time in High-Flux Hemodialysis and Hemodiafiltration

**DOI:** 10.1053/j.ajkd.2016.10.025

**Published:** 2017-06

**Authors:** James R. Smith, Norica Zimmer, Elizabeth Bell, Bernard G. Francq, Alex McConnachie, Robert Mactier

**Affiliations:** 1 Glasgow Renal and Transplant Unit, Queen Elizabeth University Hospital, Glasgow, Scotland, United Kingdom; 2 Centre for Inflammation Research, University of Edinburgh, Edinburgh, Scotland, United Kingdom; 3 Robertson Centre for Biostatistics, University of Glasgow, Glasgow, Scotland, United Kingdom

**Keywords:** Hemodialysis (HD), high-flux HD, hemodiafiltration (HDF), recovery time, intradialytic hypotension, symptomatic hypotension, blood pressure, dialysis circuit clotting, dialysis modality, end-stage kidney disease (ESKD), quality of life, randomized controlled trial (RCT)

## Abstract

**Background:**

The choice between hemodiafiltration (HDF) or high-flux hemodialysis (HD) to treat end-stage kidney disease remains a matter of debate. The duration of recovery time after treatment has been associated with mortality, affects quality of life, and may therefore be important in informing patient choice. We aimed to establish whether recovery time is influenced by treatment with HDF or HD.

**Study Design:**

Randomized patient-blinded crossover trial.

**Settings & Participants:**

100 patients with end-stage kidney disease were enrolled from 2 satellite dialysis units in Glasgow, United Kingdom.

**Intervention:**

8 weeks of HD followed by 8 weeks of online postdilution HDF or vice versa.

**Outcomes:**

Posttreatment recovery time, symptomatic hypotension events, dialysis circuit clotting events, and biochemical parameters.

**Measurements:**

Patient-reported recovery time in minutes, incidence of adverse events during treatments, hematology and biochemistry results, quality-of-life questionnaire.

**Results:**

There was no overall difference in recovery time between treatments (medians for HDF vs HD of 47.5 [IQR, 0-240] vs 30 [IQR, 0-210] minutes, respectively; *P* = 0.9). During HDF treatment, there were significant increases in rates of symptomatic hypotension (8.0% in HDF vs 5.3% in HD; relative risk [RR], 1.52; 95% CI, 1.2-1.9; *P* < 0.001) and intradialytic tendency to clotting (1.8% in HDF vs 0.7% in HD; RR, 2.7; 95% CI, 1.5-5.0; *P* = 0.002). Serum albumin level was significantly lower during HDF (3.2 vs 3.3 g/dL; *P* < 0.001). Health-related quality-of-life scores were equivalent.

**Limitations:**

Single center; mean achieved HDF convection volume, 20.6 L.

**Conclusions:**

Patients blinded to whether they were receiving HD or HDF in a randomized controlled crossover study reported similar posttreatment recovery times and health-related quality-of-life scores.

End-stage kidney disease has a significant and deleterious impact on duration and quality of life.^1^, ^2^, ^3^, ^4^ Approximately 1.9 million patients receive renal replacement therapy worldwide.^5^ Intermittent renal replacement therapy remains essential for many, and extracorporeal treatments for end-stage kidney disease such as hemodialysis (HD) and hemodiafiltration (HDF) have a higher incidence and prevalence than peritoneal dialysis, particularly in the developed world.^6^, ^7^, ^8^

Observational data have suggested that HDF is beneficial. However, randomized controlled trial (RCT) data comparing HDF with HD have produced mixed results, with analyses (mainly post hoc) suggesting that HDF has superior cardiovascular and mortality outcomes limited to patients receiving the highest convection volumes.^9^, ^10^, ^11^, ^12^, ^13^, ^14^ Although this is encouraging, high convection volumes may not be achievable in HDF patients who have suboptimal vascular access and/or time constraints associated with real-life dialysis provision.^15^

Factors influencing patient preference and choice are becoming more prominent. Patients treated with HD have lower health-related quality-of-life (HRQoL) scores than the general population, and this is associated with increased morbidity and mortality.^16^, ^17^ Although some previous studies have shown an improvement in HRQoL with convective treatments compared to HD,^3^, ^4^ the largest RCT to have studied this outcome found no difference between HDF and low-flux HD.^9^ Length of recovery time after dialysis is an important patient-reported outcome measure that adversely affects HRQoL, and evidence from the Dialysis Outcomes and Practice Patterns Study (DOPPS) cohort has suggested an association between longer postdialysis recovery time and increased mortality.^18^

We performed a patient-blinded randomized crossover study of patient-reported recovery time to determine whether recovery time differs between HD and HDF.

## Methods

### Study Design

A patient-blinded, randomized, controlled, crossover design was used. Patients were randomly assigned in a 1:1 ratio to receive 8 weeks of HD followed immediately by 8 weeks of online postdilution HDF, or vice versa. Patients were recruited from 2 satellite dialysis units (Stobhill Hospital and Glasgow Royal Infirmary) and consented by E.B., N.Z., J.R.S., or R.M. The study ran from July 2013 through March 2014. Randomization was conducted by E.B. using a remote telephone-based system run by the Robertson Centre for Biostatistics, University of Glasgow, United Kingdom.

The study was conducted in accordance with the ethics principles of the Declaration of Helsinki; all patients provided written informed consent. Good Clinical Practice guidelines were followed throughout. The West of Scotland Research and Ethics Committee approved the study (13/WS/0010). Anonymized data were sent to the Robertson Centre for Biostatistics for analysis. Data analysis programs were developed prior to the release of randomization codes to the study statistician.

### Patient Selection

There were 198 patients screened for eligibility. Inclusion criteria were receiving HD for more than 90 days, reliable vascular access, and age of 18 years or older. Exclusion criteria were currently receiving HDF (however, no recruited patients ended up having previous HDF exposure), life expectancy less than 6 months, active neoplasia, recent (within 1 month) emergency hospital admission, and unable to give informed consent or complete questionnaires. Of 119 patients meeting these criteria, 100 underwent randomization, stratified by age (4 strata: 18-49, 50-59, 60-69, and ≥70 years) and sex (given sex differences noted in recovery time reporting^18^) into one of 2 groups: HD followed by HDF, or HDF followed by HD. A separate randomization list was generated for each stratum by a computer program, using the method of randomized permuted blocks of length 4.

### Treatments

Patients received 3 treatment sessions per week. Dialysis time, dialyzer blood flow rate, dialysate flow rate, postdialysis weight, and medications were kept constant unless changes were required on clinical grounds. High-flux dialyzers (FX80 or FX100; Fresenius) were used to remain consistent with patients’ previous dialyzers and reduce the risk for inadvertent unblinding. Dialysate composition was as follows: sodium, 138 mmol/L; potassium, 2 mmol/L; chloride, 108.5 mmol/L; bicarbonate, 32 mmol/L; acetate, 3 mmol/L; calcium, 1.25 mmol/L; magnesium, 0.5 mmol/L; and glucose, 1 g/L. Fresenius 5008 dialysis machines were used. To further ensure patient blinding, dialysis machines were turned away and the on-screen treatment modality notification was covered. Dialysis unit staff were not blinded to treatment allocation.

### Outcomes

The primary outcome was reported recovery time in minutes. On arrival for each session, patients were asked by the treating nurse to state how long it took them to recover completely from the preceding session. Secondary outcomes were frequency of symptomatic hypotension; frequency of intradialytic clotting events; pre–dialysis session serum concentrations of potassium, phosphate, vitamin B_12_, parathyroid hormone, β_2_-microglobulin, betaine, and interleukin 6; and Kt/V of urea.

### Measurement Methods

Predialysis hematologic and biochemical tests were performed following a 1-day treatment gap. Blood results from the middle and end point of each treatment period were used in the analysis. The nurses administering treatments were responsible for documenting primary and secondary end point data, as well as routine dialysis session duties and data collection.

Although not prespecified outcomes, the frequency of other adverse events (documented in free-text format by nursing staff), change in quality-of-life scores (Kidney Disease Quality of Life–Short Form [KDQOL-SF], version 1.3^19^), change in dialysis dose, and patients’ preferred dialysis modality at the end of the study were also recorded. This was overseen by a dedicated research nurse, who also administered the KDQOL-SF, version 1.3, questionnaires and processed blood samples.

### Sample Size Calculation

This was based on pilot data demonstrating the variation in recovery times in 100 patients over 3 consecutive HD sessions. To detect a 20% absolute reduction in recovery time with 90% power, we calculated that 82 patients would need to complete the study (41 in each group). We planned to randomly assign 100 patients in total, allowing for a dropout rate of 18%.

### Statistical Analyses

Analyses were performed on an intention-to-treat basis. Baseline characteristics and Charlson comorbidity scores^20^ were compared between groups (HD then HDF vs HDF then HD) by Fisher exact tests for categorical variables and *t* tests for continuous variables (or Wilcoxon tests if needed). Given that the distribution of recovery times was bimodal with a peak at zero, recovery times were analyzed by crossover analysis with 2 mixed models. A generalized linear mixed model (logistic regression with random effect) was run to model the odds for a patient to recover immediately (recovery time = 0 minutes) and a mixed model was run to model the delayed (recovery time > 0 minutes) recovery times (after a logarithmic transformation). The models were combined by Monte Carlo simulations, then a parametric bootstrap method was used to obtain the overall *P* value.^21^ Correlation between recovery times across successive sessions were modeled with an autoregressive model of order 1 (in which each recovery time is dependent on the previous session, and so on) by treatment and time period. Additional analyses were run for blood tests, dialysis data, Kt/V, and mean blood flow. Hypotension, clotting, and adverse events were compared between the treatments by relative risk (RR), and in order to take into account the crossover design, odds ratios (ORs) obtained by logistic regression (with random effects) were also calculated. The KDQOL survey was compared between treatments and baseline with Friedman tests.

## Results

### Study Participants

**Figure 1:**
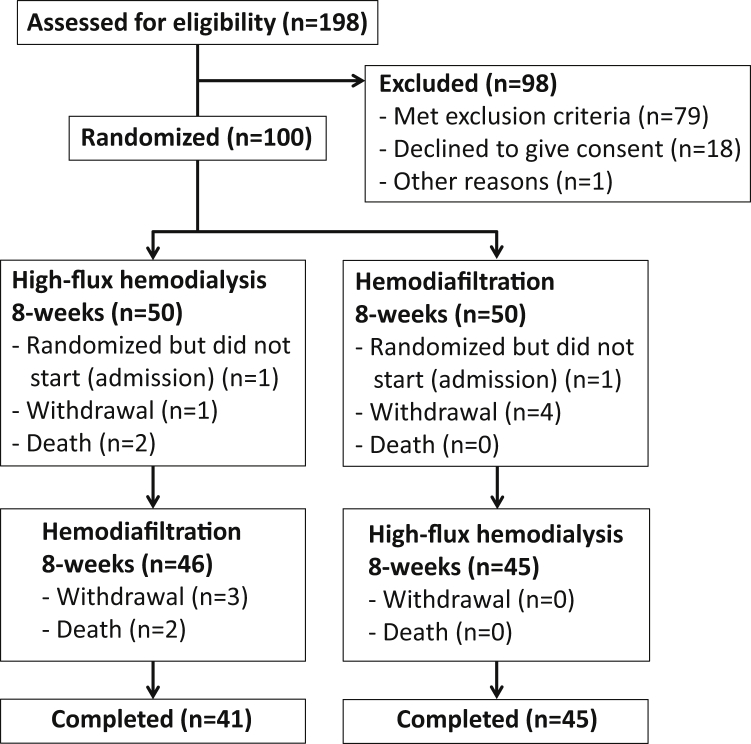
CONSORT (Consolidated Standards of Reporting Trials) flow diagram.

Table 1 shows baseline characteristics of 100 patients randomly assigned to receive HD then HDF or HDF then HD. Characteristics were generally representative of a typical prevalent HD cohort in the United Kingdom.^22^ Figure 1 gives an outline of the study flow.

**Table 1:** Baseline Characteristics of 100 Participants Randomly Assigned to HD Followed by HDF or Vice Versa

Characteristic	All (N = 100)	HD Then HDF (n = 50)	HDF Then HD (n = 50)	*P*^a^
Female sex	39 (39)	20 (40)	19 (38)	0.9
Age, y	65 ± 14	65 ± 15	66 ± 13	0.6
Dialysis vintage, mo	27 [15-63]	38 [14-90]	27 [16-46]	0.3^b^
Access				0.2
Fistula	68 (68)	31 (62)	37 (74)	
Graft	1 (1)	0 (0)	1 (2)	
Central venous catheter	31 (31)	19 (38)	12 (24)	
Antihypertensive medication use	66 (66)	31 (62)	35 (70)	0.5
SBP, mm Hg^c^	143 ± 20	144 ± 22	142 ± 18	0.7
DBP, mm Hg^c^	69 ± 12	67 ± 12	70 ± 11	0.3
Post-HD weight, kg^c^	74 [62-87]	76 [64-88]	71 [61-86]	0.2^b^
UF volume, mL^c^	1,819 ± 694	1,995 ± 681	1,644 ± 668	0.01
Primary renal diagnosis				0.7
Glomerular	26 (26)	13 (26)	13 (26)	
Tubulointerstitial	11 (11)	5 (10)	6 (12)	
Systemic	30 (30)	17 (34)	13 (26)	
Hereditary	10 (10)	3 (6)	7 (14)	
Miscellaneous	23 (23)	12 (24)	11 (22)	
Waitlisted for transplant	21 (21)	13 (26)	8 (16)	0.3
White	99 (99)	49 (98)	50 (100)	0.5
Smoking status				0.5
Current smoker	27 (27)	11 (22)	16 (32)	
Ex-smoker	30 (30)	17 (34)	13 (26)	
Never smoked	43 (43)	22 (44)	21 (42)	
Diabetes	26 (26)	15 (30)	11 (22)	0.5
Ischemic heart disease	37 (37)	20 (40)	17 (34)	0.7
Peripheral vascular disease	19 (19)	14 (28)	5 (10)	0.04
Stroke	17 (17)	10 (20)	7 (14)	0.6
History of neoplasia	7 (7)	6 (12)	1 (2)	0.1
Charlson comorbidity score	7 ± 2	7 ± 2	6 ± 2	0.4^b^

*Note:* Values for categorical variables are given as count (percentage); values for continuous variables, as mean ± standard deviation or, in the case of non-normally distributed data, median [interquartile range].

Abbreviations: DBP, diastolic blood pressure; HD, high-flux hemodialysis; HDF, hemodiafiltration; SBP, systolic blood pressure; UF, ultrafiltration.

a *P* values derived from *t* test for continuous variable (or ^b^Wilcoxon) and Fisher exact test for categorical variables; 100% of data is reported for all variables.

c Mean of 3 months of dialysis data prior to randomization.

### Clinical Treatment Parameters

Treatment time and blood flow rate remained constant between HD and HDF (Table 2). Additionally, ultrafiltration volumes were similar and the mean convection volume for HDF treatments was 20.6 L. Pretreatment systolic blood pressure (SBP) was lower while participants were receiving HDF (143 vs 145 mm Hg; *P* *=* 0.03); however, this difference was not demonstrated posttreatment (Table 2).

**Table 2:** Delivered Treatment and Peritreatment Blood Pressure: HD Versus HDF

Variable	HD	HDF	Crossover *P*	Data Available, %
Session length, min	250 ± 17	250 ± 17	0.1	94
Blood flow rate, mL/min	315 ± 27	313 ± 28	0.6	93
Ultrafiltration volume, mL	1,749 ± 718	1,723 ± 672	0.4	94
Convection volume, L	NA	20.6 ± 4.6	NA	82
Pretreatment SBP, mm Hg	145 ± 21	143 ± 21	0.03	93
Pretreatment DBP, mm Hg	69 ± 12	69 ± 12	0.1	93
Posttreatment SBP, mm Hg	126 ± 20	125 ± 18	0.08	93
Posttreatment DBP, mm Hg	64 ± 10	63 ± 10	0.1	93

*Note:* Unless otherwise indicated, values are given as mean ± standard deviation.

Abbreviations: DBP, diastolic blood pressure; HD, high-flux hemodialysis; HDF, hemodiafiltration; NA, not applicable; SBP, systolic blood pressure.

### Primary Outcome: Posttreatment Recovery Time of HDF Versus HD

**Figure 2:**
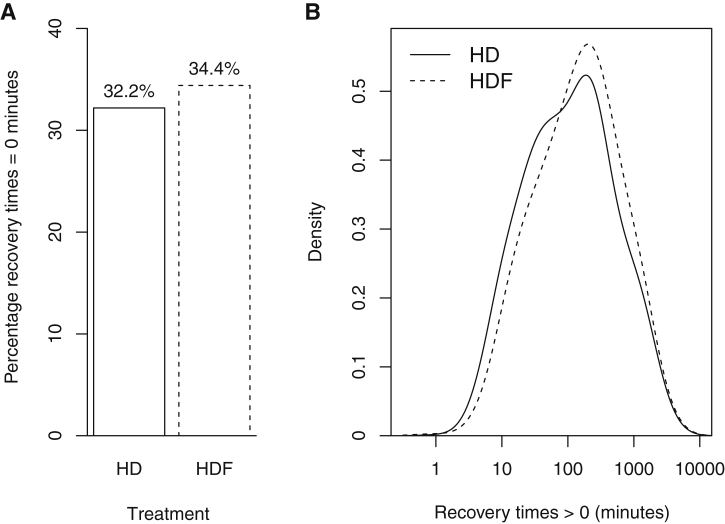
Bar and density plots of immediate and delayed recovery times for high-flux hemodialysis (HD) and hemodiafiltration (HDF). (A) Bar plot shows percentage of recovery times that were recorded as equal to zero minutes. (B) Density plot shows delayed (>0 minute) recovery times for HD and HDF. X-axis presented on log 10 scale.

Recovery time data were available for 92% of all sessions. Recovery time for one-third of available sessions was reported as zero minutes (immediate), resulting in a bimodal distribution. To account for this, immediate and delayed (>0 minutes) recovery times were analyzed using separate models, then joined to obtain an overall *P* value, as described in the Statistical Analyses section in Methods. This demonstrated no overall difference in recovery time between HDF and HD (median values of 47.5 [IQR, 0-240] and 30 [IQR, 0-210] minutes, respectively; *P* *=* 0.9). However, of interest, individual models for immediate and delayed recovery time showed that patients were more likely to report immediate recovery while receiving HDF treatment (34.4% for HDF vs 32.2% for HD; OR, 1.37; 95% confidence interval [CI], 1.08-1.74; *P* *=* 0.01; Fig 2A). Conversely, HDF resulted in longer delayed recovery times (for HDF vs HD, medians of 150 [IQR, 60-420] vs 137 ± 4.3 minutes, geometric means of 120 [IQR, 30-270] vs 104 ± 4.6 minutes, respectively; *P* < 0.001; Fig 2B). Table S1 (provided as online supplementary material) shows baseline characteristics comparing patients who reported at least one immediate recovery time with those who reported only delayed recovery times.

There was no interaction between treatments and the order in which they were given (treatment effect) for immediate (*P* = 0.8) or delayed (*P* = 0.6) recovery times. Although there was no evidence of a difference in recovery time reporting between the first and second period of the study (time effect) for delayed recovery times (OR for delayed recovery, second vs first study period, 0.99; 95% CI, 0.89-1.09; *P* = 0.8), patients were more likely to report immediate recovery during the first half of the study (OR for immediate recovery, second vs first study period, 0.77; 95% CI, 0.61-0.98; *P* *=* 0.04), regardless of treatment (Tables S2 and S3).

A washout period was not possible given the nature of the treatments. However, with regard to carryover effect, the correlation between sessions decreased quickly and was <5% for both treatments after 9 sessions. To test for the influence of carryover effect, analyses were repeated by removing up to and including the first 9 sessions of each treatment. Immediate, delayed, and overall recovery times remained equivalent after these alterations (Table S4).

### Secondary Outcomes

#### Symptomatic Hypotension

HDF was associated with an increased rate of symptomatic hypotension compared to HD (8.0% vs 5.3%; RR, 1.52; 95% CI, 1.2-1.9; *P* < 0.001; Table 3). Antihypertensive dosing was increased in 3 patients while on HD therapy and 1 patient while on HDF therapy (*P* *=* 0.6); similarly, dosing was reduced in 3 patients while on HD therapy and 1 patient while on HDF therapy (*P* *=* 0.6).

**Table 3:** Adverse Events for HD and HDF, Per Session

Variable	HD Sessions	HDF Sessions	RR (95% CI)	*P*	Data Available, %
Symptomatic hypotension^a^	112 (5.2)	168 (8)	1.52 (1.21-1.92)	<0.001	97
AEs potentially related to BP/fluid shifts^b^	61 (3.0)	109 (5.3)	1.81 (1.33-2.46)	<0.001	93
AEs not classically related to BP/fluid shifts^c^	88 (4.3)	87 (4.3)	1.00 (0.75-1.34)	0.9	93
Extra tinzaparin dose(s) or clotting of circuit^d^	14 (0.7)	37 (1.8)	2.68 (1.46-5.00)	0.002	97

*Note:* Unless otherwise indicated, values are given as number of events (percentage). Multiple episodes within 1 session were treated as a single event. Odds ratios taking into account the crossover design were also calculated and were almost identical to RRs.

Abbreviations: AE, adverse event; BP, blood pressure; CI, confidence interval; HD, high-flux hemodialysis; HDF, hemodiafiltration; RR, relative risk.

a Defined as a decrease in systolic BP ≥ 20 mm Hg requiring reduction or cessation of ultrafiltration and/or need for intravenous fluid bolus or head-down tilt of dialysis chair.

b Breathlessness, cramp (normal BP), dizzy/lightheaded, fall, headache, venous pressures erratic, clotted needle, or restless legs.

c Aches in bones, arm pain, back pain, bleeding, constipation, diarrhea, feeling cold, feeling down, feeling hot, generally unwell, heavy legs, increased lethargy, infection (given antibiotics), itch, leg pain, nausea, stomach pains, sweating, swollen abdomen, and vomiting.

d Defined as either an increase in venous pressure requiring additional anticoagulant dosing or clotting of the extracorporeal circuit.

#### Clotting Risk

The intradialytic tendency to clotting was higher during HDF than HD (1.8% vs 0.7%; RR, 2.7; 95% CI, 1.5-5.0; *P* = 0.002; Table 3). There was no difference in tinzaparin dosing (2,773 ± 147 and 2,740 ± 140 units for HDF and HD, respectively; *P* = 0.9).

#### Laboratory Values

None of the prespecified laboratory measurements showed statistically significant differences between HDF and HD treatments (Table 4). Blood samples were stored for additional analyses (eg. β2-microglobulin, betaine, interleukin 6) but these samples have not been processed.

**Table 4:** Midweek Pretreatment Blood Test Results and Urea Clearance, HD Versus HDF

Variable	HD	HDF	Crossover *P*	Data Available, %
Hemoglobin, g/dL	11.5 ± 1.3	11.3 ± 1.3	0.1	92
WBC count, ×10^3^/μL	7.3 ± 2.6	7.4 ± 2.7	0.5	92
Platelets, ×10^3^/μL	225 ± 73	226 ± 79	0.7	92
C-Reactive protein,^a^ mg/L	14 ± 17	12 ± 10	0.9	92
Calcium, mg/dL	9.6 ± 0.4	9.6 ± 0.8	0.4	92
Phosphate, mg/dL	5.0 ± 1.2	5.0 ± 1.2	0.7	92
PTH, pg/mL	701 ± 483	666 ± 474	0.2	88
Albumin, g/dL	3.3 ± 0.3	3.2 ± 0.3	<0.001	92
Sodium, mEq/L	137 ± 2	137 ± 3	0.9	84
Potassium, mEq/L	4.8 ± 0.6	4.8 ± 0.6	0.6	84
Chloride, mEq/L	100 ± 2	101 ± 3	0.02	84
Bicarbonate, mEq/L	20 ± 2	20 ± 2	0.3	84
Urea reduction ratio	75 ± 5	76 ± 6	0.5	92
Kt/V	1.6 ± 0.4	1.7 ± 0.4	0.2	90
Creatinine, mg/dL	8.2 ± 2.3	8.2 ± 2.4	0.6	84
Vitamin B_12_,^a^ ng/L	460 ± 273	491 ± 360	0.3	89
Folate, ng/mL	23 ± 6	24 ± 6	0.5	92
Ferritin,^a^ μg/L	397 ± 240	419 ± 272	0.3	92
Reticulocytes, ×10^3^/μL	66 ± 27	71 ± 29	0.05	89

*Note:* Unless otherwise indicated, values are given as mean ± standard deviation. Conversion factors for units: calcium in mg/dL to mmol/L, ×0.2495; creatinine in mg/dL to μmol/L, ×88.4; folate in ng/mL to nmol/L, ×2.266; glucose in mg/dL to mmol/L, ×0.05551; phosphate in mg/dL to mmol/L, ×0.3229.

Abbreviations: HD, high-flux hemodialysis; HDF, hemodiafiltration; PTH, parathyroid hormone; WBC, white blood cells.

a Data log transformed prior to statistical tests.

### Other Measures

#### Other Laboratory Measures

As shown in Table 4, there were small but statistically significant differences in serum albumin (3.2 vs 3.3 g/dL for HDF and HD, respectively; *P* < 0.001) and chloride levels (101 vs 100 mEq/L for HDF and HD; *P* *=* 0.02).

#### Other Adverse Events

Other adverse events were grouped according to those that may or may not have been related to blood pressure (BP) changes or fluid shifts (Table 3). Although the frequency of events not typically associated with BP or fluid shifts was similar between groups, there were more instances of events more likely to be related to BP or fluid shifts during HDF treatment compared to HD (5.3% vs 3.0%; RR, 1.8; 95% CI, 1.3-2.5; *P* < 0.001).

#### Quality-of-Life Scores

Patients completed KDQOL-SF, version 1.3, questionnaires^19^ during the study. Patients scored physical health lower (33 ± 10) than mental health (44 ± 10) at baseline. There was no difference in physical health composite scores (33 ± 9 for both HDF and HD; *P* *=* 0.9) or mental health composite scores (44 ± 11 vs 43 ± 12 for HDF and HD, respectively; *P* *=* 0.6) after 8 weeks of each treatment.

#### Withdrawals/Deaths

There were 9 withdrawals during the study, 2 occurring after randomization but before starting the trial as a result of emergency hospital admissions. Four patients died during the study. Further details are given in Table S5.

#### Maintenance of Blinding and Patient Preferences

Blinding to treatment was maintained in 84 of the 86 patients who completed the study. While still blinded, these 84 patients were asked which treatment they preferred; 52 had no preference, 21 preferred HDF, and 11 preferred HD. Eight patients felt able to guess the treatment order based on their symptoms; 5 were correct and 3 were incorrect.

## Discussion

This patient-blinded, randomized, controlled, crossover trial showed no difference in the primary outcome of patient-reported posttreatment recovery time between HDF and HD (medians of 47.5 [IQR, 0-240] and 30 [IQR, 0-210] minutes, respectively; *P* *=* 0.9). However, recovery time data had a bimodal distribution, with more than one-third of recovery times being recorded as zero minutes (immediate). Therefore, analysis of immediate recovery was performed separately from delayed recovery, with interesting yet disparate results. Treatment with HDF was associated with a significantly higher chance of immediate recovery (*P* = 0.01), but resulted in significantly longer recovery time than HD for those who had delayed recovery (*P* < 0.001).

We hypothesize that inherent differences among patients reporting immediate and delayed recovery times may have influenced treatment tolerance. For example, patients with fewer comorbid conditions may report more symptomatic benefit from enhanced small- and middle-molecule clearance attributed to convective treatments, whereas patients with more comorbid conditions may tolerate HDF less well. A recent subanalysis of the DOPPS cohort showed that recovery time, based on a categorical measure at a single time, was similar in patients on HDF and HD therapy (OR, 1.08; 95% CI, 0.87-1.35).^18^ Interestingly, longer recovery time was associated with increased mortality, as well as adverse clinical features such as older age, higher body mass index, and diabetes.^18^ We found that patients who reported immediate recovery were significantly more likely at baseline to be lighter (73 vs 86 kg; *P* = 0.008) and active on the transplant waiting list (29% vs 6%; *P* = 0.009) than patients who reported only delayed recovery times, but other baseline characteristics were similar (Table S1).

Patient-reported posttreatment recovery time is inherently subjective, difficult to measure robustly, and at risk for reporting or recall bias. Our choice of crossover design was made in the face of these potential issues in order to abrogate the influence of interpatient variability. The question “How long does it take you to recover from a dialysis session” has been validated as easy to understand and stable in the face of retest, yet sensitive to alterations in treatment,^23^ and this was the same question administered to the DOPPS cohort.^18^ The former study asked the question at 3-month intervals (recording the answer as a continuous variable), and in the latter study, the question was asked once at the beginning of the study and collected as a categorical variable. In order to achieve sufficient granularity, we recorded recovery time as a continuous variable; how this compares to a categorical approach is not known. In addition, we adapted the question asked of the study patients to “How long did it take you to recover from your previous treatment session” given that this was being asked at every session. The differences noted between treatment modalities in immediate and delayed recovery time reporting in this study suggest that this approach was sufficiently sensitive to detect changes in recovery time.

We found no difference in HRQoL scores between HD and HDF, which is consistent with the result of the overall primary outcome. Longer postdialysis recovery time has been associated with poorer HRQoL scores.^18^, ^23^ Additionally, associations have also been drawn between lower HRQoL scores and patient mortality.^17^, ^24^, ^25^

There was an increase in risk for intradialytic clotting with HDF, which in the majority of cases was mitigated by an increase in anticoagulant dose prior to clotting of the circuit. Clotting resulting in the need to change the circuit was infrequent, occurring in 0.62% of HDF sessions and 0.38% of HD sessions. Postdilution HDF provides more efficient convective clearance compared with predilution HDF, but has previously been associated with an increase in clotting events compared to HD.^26^ There was no difference in mean anticoagulant doses between treatments in our study, perhaps due to the small number of clotting events prompting an increase in subsequent anticoagulant doses.

Unexpectedly, we found that HDF was associated with increased symptomatic hypotension. Eighty percent of these episodes were classed as mild, requiring only cessation of ultrafiltration. There was also a small reduction in pretreatment SBP in those receiving HDF, whereas posttreatment SBP was equivalent between treatments. Convective treatments are often considered to have a hemodynamic advantage over HD. This has been described in studies comparing low-flux HD to hemofiltration or HDF,^27^, ^28^ but not in a study in which both modalities were performed under matched conditions,^29^ and we are not aware of blinded trials analyzing this outcome. When comparing HD to HDF, Ok et al^10^ found no difference in symptomatic hypotension (defined as a decrease in SBP > 30 mm Hg requiring saline solution infusion), and in the ESHOL (Estudio de Supervivencia de Hemodiafiltración On-Line) study, the incidence of symptomatic hypotension (not clearly defined) was lower in the HDF group (rate ratio, 0.72; 95% CI, 0.68-0.77; *P* < 0.001).^13^ The reasons behind the disparate findings are unclear, but importantly, in our study, there were no significant changes in antihypertensive treatment, ultrafiltration volume, blood flow, or dialysis dose between treatments.

With regard to blood parameters, it is worth highlighting the small reduction in serum albumin levels during HDF treatment compared to HD (3.2 vs 3.3 g/dL, respectively; *P* < 0.001). Data for serum albumin levels in previous studies comparing HDF to HD are variable. Ok et al^10^ found that albumin levels were lower in the HDF group, whereas in ESHOL, there was no difference between treatments.^13^ However, serum albumin levels decreased in both groups compared to baseline over the course of both these randomized studies.^10^, ^13^ Even small reductions in serum albumin levels are strongly associated with reduced survival in HD patients.^30^ Therefore, it is important to be aware of such changes, particularly in the presence of pre-existing hypoalbuminemia.

In light of these findings, it is conceivable that more frequent episodes of hypotension and lower serum albumin levels may have influenced recovery time adversely in the HDF group. However, we found no difference in results of the primary outcome when adjustments were made for BP and serum albumin level (Table S4).

The success of patient blinding exceeded our expectations and is worthy of note; this should be considered a realistic strategy for future studies investigating extracorporeal treatments. Due to the practicalities of administering these treatments, double-blinding was not possible, and the treating nurses were responsible for collecting end point data from patients. However, statistical analyses were performed on anonymized allocation-blinded data. Inclusion criteria were designed to be as broad as possible to serve as a representative cohort of long-term HD patients. It may be that this in part limited our ability to achieve HDF convection volumes described in previous studies. For example, in our study, the arteriovenous fistula rate (68%) was lower than in the ESHOL online-HDF group (89%), as were mean blood flow rates (313 mL/min in our study vs 389 mL/min after 36 months in the ESHOL online-HDF group).^13^ These factors, in addition to treatment time, are known to be important in determining achieved convection volumes.^15^, ^31^ In our study, mean treatment time was 250 minutes, longer than the 237 minutes reported after 36 months in the ESHOL online-HDF group,^13^ suggesting that blood flow rate was one of the most important factors limiting achieved convection volume.

The single-center (although multiple satellite unit) design is a limitation. Additionally, the predominance of patients of European ancestry, although representative of our case-mix, will be less applicable in other regions. It is also possible that the nursing staff’s longer term experience with HD compared to HDF could have had an influence on results. However, this situation is likely to be the case in most nephrology units, and crossover analyses demonstrated more immediate recovery times for both treatments during the first treatment period, with no difference in delayed recovery times between treatment periods, suggesting that increasing staff experience in delivering HDF treatment is likely to have had minimal influence.

To our knowledge, this is the first RCT to examine recovery time in the setting of HD and HDF, and we found no overall difference between treatments. However, HDF was associated with both more episodes of immediate recovery and longer recovery time if recovery was not immediate. Although more recent data may suggest a beneficial role for online HDF with high convection volumes, many patients may be unable to achieve such convection volumes and therefore neither treatment is definitively superior for all patients.^32^ The overall result of our primary outcome is consistent with the findings of previous observational studies^18^, ^24^, ^25^; however, it is difficult to infer which of the many variables associated with extracorporeal treatments may be responsible for effects on recovery time or mortality. For example, more frequent (6 times per week) HD has been associated with a reduction in recovery time compared to thrice-weekly HD,^33^ as well as improvements in areas such as BP and serum phosphate control, left ventricular mass, and HRQoL.^34^, ^35^, ^36^ Whether this leads to improvements in patient survival remains a point of debate.^37^, ^38^, ^39^, ^40^ With this in mind and in light of the association between recovery time and mortality as identified in the DOPPS cohort,^18^ a large-scale, blinded, multicenter RCT of HDF and HD powered to examine morbidity and mortality outcomes while taking into account recovery time reporting and quality-of-life scores across a representative patient group would be of benefit.

Debate remains regarding the clinical case for HDF over HD. Patient preference and shared decision making are increasingly prioritized in clinical practice, and these data may further inform the discussion around choice of extracorporeal treatments.

## Supplementary Material

Supplementary Table S1 (PDF) Baseline characteristics of participants reporting ≥1 immediate recovery time vs none.

Supplementary Table S2 (PDF) Percentage of immediate recovery times according to treatment sequence.

Supplementary Table S3 (PDF) Delayed recovery times according to treatment sequence.

Supplementary Table S4 (PDF) Sensitivity analysis of recovery times: comparison of delayed and immediate times (HDF vs HD).

Supplementary Table S5 (PDF) Withdrawals and deaths during study period.
