# Supplementary material for: A Randomized, Single-Blind, Crossover Trial of Recovery Time in High-Flux Hemodialysis and Hemodiafiltration
Source: Am J Kidney Dis. 2017 Jun;69(6):762–70. doi: 10.1053/j.ajkd.2016.10.025 (PMC5438239; doi:10.1053/j.ajkd.2016.10.025)
Supplement: Supplementary Table S1 (PDF) — Baseline characteristics of participants reporting ≥1 immediate recovery time vs none. [file mmc1.pdf]

Table S1 – Baseline characteristics of study participants reporting at least one immediate recovery time vs. no immediate recovery times

Characteristic	All n=97	At least one RT = 0 n=65	No RT = 0 n=32	*p* value
**Female**	39 (40)	29 (45)	10 (31)	0.3
**Age (years)**	65 ± 14	64 ± 14	68 ± 13	0.2
**Dialysis vintage (months) (median [IQR])**	27 [15, 63]	25 [15, 60]	36 [16, 67]	0.5[b]
**Access**				0.8
Fistula	65 (67)	42 (65)	23 (72)	
Graft	1 (1)	1 (2)	0 (0)	
Central Venous Catheter	31 (32)	22 (34)	9 (28)	
**SBP (mmHg)[a]**	143 ± 20	142 ± 20	144 ± 21	0.6
**DBP (mmHg)[a]**	69 ± 12	67 ± 13	72 ± 10	**0.04**
**Post HD weight (kg)[a] (median [IQR])**	74 [62, 87]	70 [61, 83]	84 [68, 93]	**0.01[b]**
**UF volume (ml)[a]**	1819 ± 704	1833 ± 708	1788 ± 706	0.8
**Primary renal diagnosis**				0.6
Glomerular	25 (26)	14 (22)	11 (34)	
Tubulointerstitial	11 (11)	8 (12)	3 (9)	
Systemic	29 (30)	21 (32)	8 (25)	
Hereditary	9 (9)	5 (8)	4 (12)	
Miscellaneous	23 (24)	17 (26)	6 (19)	
**Transplant listed**	21 (22)	19 (29)	2 (6)	**0.009**
**Smoking**				0.9
Current	26 (27)	18 (28)	8 (25)	
Ex-smoker	30 (31)	19 (29)	11 (34)	
Never smoked	41 (42)	28 (43)	13 (41)	
**Diabetes**	26 (27)	18 (28)	8 (25)	1
**Ischemic heart disease**	36 (37)	24 (37)	12 (38)	1
**Peripheral vascular disease**	19 (20)	12 (18)	7 (22)	0.8
**Stroke**	17 (18)	13 (20)	4 (12)	0.4
**History of neoplasia**	7 (7)	6 (9)	1 (3)	0.4
**Charlson comorbidity score**	7 ± 2	7 ± 3	6 ± 2	0.3[b]

Note: Values given as number (percentage) or mean ± standard deviation, or in the case of non-normally distributed data, median [IQR]. Patients who withdrew from the study before reporting any recovery time data are excluded (n=3).
Abbreviations: RT, Recovery time; HD, high-flux hemodialysis; HDF, hemodiafiltration; SBP, systolic blood pressure; DBP, diastolic blood pressure; HD, hemodialysis; UF, ultrafiltration. [a]Mean of three months of dialysis data prior to randomization. P values derived from t-test for continuous variable (or Wilcoxon[b]) and Fisher's Exact-test for categorical variables. 100% data is reported for all variables.
