# Supplementary material for: A Randomized, Single-Blind, Crossover Trial of Recovery Time in High-Flux Hemodialysis and Hemodiafiltration
Source: Am J Kidney Dis. 2017 Jun;69(6):762–70. doi: 10.1053/j.ajkd.2016.10.025 (PMC5438239; doi:10.1053/j.ajkd.2016.10.025)
Supplement: Supplementary Table S2 (PDF) — Percentage of immediate recovery times according to treatment sequence. [file mmc2.pdf]

Table S2 – Percentage of immediate (=0 minutes) recovery times according to treatment sequence

**Allocated Treatment Sequence**	**All sessions**	**HD sessions**	**HDF sessions**
**HD then HDF (n=1980)**	32.9%	32.5%	33.3%
**HDF then HD (n=2067)**	33.7%	31.9%	35.4%

Abbreviations: HD, high-flux hemodialysis; HDF, hemodiafiltration.
