# Supplementary material for: A Randomized, Single-Blind, Crossover Trial of Recovery Time in High-Flux Hemodialysis and Hemodiafiltration
Source: Am J Kidney Dis. 2017 Jun;69(6):762–70. doi: 10.1053/j.ajkd.2016.10.025 (PMC5438239; doi:10.1053/j.ajkd.2016.10.025)
Supplement: Supplementary Table S3 (PDF) — Delayed recovery times according to treatment sequence. [file mmc3.pdf]

Table S3 – Delayed (>0 minutes) recovery times according to treatment sequence

Allocated Treatment Sequence	HD sessions median [IQR]	HDF sessions median [IQR]
**HD then HDF (mins)**	90 [30, 300]	150 [60, 395]
**HDF then HD (mins)**	120 [30, 240]	150 [60, 420]

Abbreviations: HD, high-flux hemodialysis; HDF, hemodiafiltration; mins, minutes; IQR, Inter-quartile range.
