# Supplementary material for: A Randomized, Single-Blind, Crossover Trial of Recovery Time in High-Flux Hemodialysis and Hemodiafiltration
Source: Am J Kidney Dis. 2017 Jun;69(6):762–70. doi: 10.1053/j.ajkd.2016.10.025 (PMC5438239; doi:10.1053/j.ajkd.2016.10.025)
Supplement: Supplementary Table S4 (PDF) — Sensitivity analysis of recovery times: comparison of delayed and immediate times (HDF vs HD). [file mmc4.pdf]

Table S4 – Sensitivity analysis of recovery times: Comparison of delayed recovery times and immediate recovery times (HDF vs. HD), excluding up to 9 initial sessions to account for carry-over effect.

	Immediate recovery time		Delayed recovery time		
**Number of sessions**	**HDF vs. HD Odds Ratio (95% CI)**	***p* value**	**HDF vs. HD - Ratio of geometric means (95% CI)**	***p* value**	**Overall (delayed + immediate) *p* value**
All 24[a]	1.37 (1.08, 1.74)	0.01	1.23 (1.11,1.37)	<0.001	0.9
All 24[b]	1.38 (1.06, 1.79)	0.02	1.29 (1.14, 1.45)	<0.001	
Last 21	1.54 (1.18, 2.00)	0.001	1.19 (1.07, 1.33)	0.002	0.9
Last 18	1.61 (1.20, 2.17)	0.002	1.20 (1.07, 1.34)	0.002	0.9
Last 15	1.50 (1.08, 2.07)	0.02	1.2 (1.05, 1.37)	0.009	0.9

Abbreviations: HD, high-flux hemodialysis; HDF, hemodiafiltration; CI, confidence interval.
[a]Unadjusted, [b]adjusted for episodes of symptomatic hypotension, pre-treatment systolic blood pressure and serum albumin.
