# Supplementary material for: A Randomized, Single-Blind, Crossover Trial of Recovery Time in High-Flux Hemodialysis and Hemodiafiltration
Source: Am J Kidney Dis. 2017 Jun;69(6):762–70. doi: 10.1053/j.ajkd.2016.10.025 (PMC5438239; doi:10.1053/j.ajkd.2016.10.025)
Supplement: Supplementary Table S5 (PDF) — Withdrawals and deaths during study period. [file mmc5.pdf]

Table S5 - Withdrawals and deaths during the study period

**Age (years)**	**Treatment period**	**Reason for withdrawal**
56	HFHD	Anxiety about fluid balance/restriction
64	HDF	Erratic venous pressures, patient anxious about fistula patency
77	HDF	Transferred to another unit
68	HDF	Persistent cramps
80	HDF	Persistent episodes of hypotension during treatment
43	HDF	No reason given
44	HDF	Increased headaches
**Age (years)**	**Treatment Period**	**Cause of death**
68	HDF	Sudden cardiac death
62	HFHD	Myocardial infarction
86	HDF	Sepsis
84	HFHD	Intra-abdominal sepsis

Abbreviations: HFHD, high-flux hemodialysis; HDF, hemodiafiltration
